# HybriFree: a robust and rapid method for the development of monoclonal antibodies from different host species

**DOI:** 10.1186/s12896-016-0232-6

**Published:** 2016-01-08

**Authors:** Gaily Kivi, Kaupo Teesalu, Jüri Parik, Elen Kontkar, Mart Ustav, Liis Noodla, Mart Ustav, Andres Männik

**Affiliations:** Icosagen Cell Factory OÜ, Eerika tee 1, Õssu village, Ülenurme parish, Tartumaa, 61713 Estonia; University of Tartu, Institute of Technology, Nooruse1, Tartu, 50411 Estonia; Estonian Biocentre, Evolutionary Biology Group, Tartu, 51010 Estonia; Estonian Academy of Sciences, Kohtu 6, Tallinn, 10130 Estonia

**Keywords:** Recombinant antibodies, Mammalian cell based screening mammalian cell culture, Protein production

## Abstract

**Background:**

The production of recombinant monoclonal antibodies in mammalian cell culture is of high priority in research and medical fields. A critical step in this process is the isolation of the antigen-binding domain sequences of antibodies possessing the desired properties. Many different techniques have been described to achieve this goal, but all have shortcomings; most techniques have problems with robustness, are time-consuming and costly, or have complications in the transfer from isolation to production phase. Here, we report a novel HybriFree technology for the development of monoclonal antibodies from different species that is robust, rapid, inexpensive and flexible and can be used for the subsequent production of antibodies in mammalian cell factories.

**Results:**

HybriFree technology is illustrated herein via detailed examples of isolating mouse, rabbit and chicken monoclonal antibody sequences from immunized animals. Starting from crude spleen samples, antigen capturing of specific B-cells is performed initially. cDNA of antibody variable domains is amplified from the captured cells and used a source material for simple and rapid restriction/ligation free cloning of expression vector library in order to produce scFv-Fc or intact IgG antibodies. The vectors can be directly used for screening purposes as well as for the subsequent production of the developed monoclonal antibodies in mammalian cell culture. The antibodies isolated by the method have been shown to be functional in different immunoassays, including ELISA, immunofluorescence and Western blot. In addition, we demonstrate that by using a modified method including a negative selection step, we can isolate specific antibodies targeting the desired epitope and eliminate antibodies directed to undesired off-targets.

**Conclusions:**

HybriFree can be used for the reliable development of monoclonal antibodies and their subsequent production in mammalian cells. This simple protocol requires neither the culturing of B-cells nor single-cell manipulations, and only standard molecular biology laboratory equipment is needed. In principle, the method is applicable to any species for which antibody cDNA sequence information is available.

**Electronic supplementary material:**

The online version of this article (doi:10.1186/s12896-016-0232-6) contains supplementary material, which is available to authorized users.

## Background

Monoclonal antibodies (MAbs) are made by identical immune cells and target one particular epitope by monovalent or monospecific affinity. The high affinity and selective binding of MAbs to epitopes in target antigens makes them highly potent tools for use in biochemistry, molecular biology and medicine. The first working method described for the isolation of monoclonal antibodies was hybridoma technology, based on forming hybrid cell lines (hybridomas) by fusing an antibody-producing B-cell with a myeloma cell [1]. The antibodies produced by a particular hybridoma clone share the same specificity. Thus, individual clones can be screened for the production of an antibody with the desired affinity. However, hybridoma technology has shortcomings: it takes a relatively long time (on the order of months) and has not been widely applied to organisms other than mice. Moreover, antibody sequence information is unavailable by this method. Thus, when a hybridoma-screened antibody is selected for further development (e.g., as a therapeutic product), the cDNA encoding the variable domains of the heavy (VH) and light chain (VL) must be isolated from the hybridoma cells. This step is required for the recombinant production of the final MAb product, as well as for improvements such as humanization, isotype conversion, and affinity maturation.

Alternatively, recombinant antibody isolation technologies usually do not include a hybrid cell line step, but instead clone the VH and VL domain sequences from the antibody-expressing source cells (e.g., B-cells from spleen, bone marrow or blood). Commonly, VH and VL cDNA is amplified by RT-PCR using mRNA isolated from the cells. By combinatorial strategies, a large repertoire of different VH and VL sequences are amplified from a population of cells (e.g., millions of B-cells isolated from an immunized animal). Thereafter, the amplified products are used for the construction of combinatorial libraries by the random pairing of the VH and VL domains. Thus, combinatorial strategies must involve a screening step for the identification of antibodies (VH and VL combinations) with the desired properties from large libraries. These screening methods involve in vitro antibody display techniques including phage display [2, 3], ribosome display [4], and in vivo display platforms such as bacterial, yeast, and mammalian cell-surface displays [5]. Mammalian cell display has a number of advantageous features, especially for selecting antibodies for recombinant production [6]. For example, using mammalian cells for MAb screening minimizes the loss of MAb during downstream production in mammalian cells, a complication sometimes associated with MAbs screened in phage, bacterial or even yeast display systems. Instead, performing antibody selection in mammalian cells ensures that the selected antibody is synthesized, modified, assembled and secreted through the same cellular pathways employed in the production phase.

Recombinant methods also include non-combinatorial strategies that are based on retrieving antibody sequences from a single B-cell or the clonally expanded progeny of a single cell. Using this technique, the original heavy and light chain pairs are maintained together during the isolation process (for a review, see ref. [7]). This is achieved by the amplification of rearranged VH and VL region cDNAs from one particular B-cell by technically challenging single-cell RT-PCR methods [8]. Furthermore, isolated single B-cells can be amplified in cell culture. These methods include the immortalization of differentiated B-cells by the Epstein-Barr virus (EBV), retroviral oncogenes, or TLR ligands (reviewed in ref. [7]); by cultivation under specific conditions with irradiated thymoma cells [9, 10]; or by IL4 treatment and CD40 triggering [11]. These techniques were developed to facilitate and increase the efficiency of non-combinatorial strategies (e.g., the enrichment of source B-cells that express antibodies with the desired affinity). However, all non-combinatorial methods involve either complicated single-cell manipulations and/or the establishment of sterile primary cell cultures from unsterile biological materials, as well as specialized equipment and/or expensive supplements. Any MAb development strategy can potentially include negative or “subtractive” screening steps to eliminate the recovery of antibodies recognizing the target antigen but also some non-desired off-target(s). This is especially useful for the development of very specific MAbs that can bind to specific protein modifications [12, 13] or recognize a specific member of a protein family with common domains, motifs and/or structures [14].

Here, we describe the novel HybriFree technology, which combines elements of both combinatorial and non-combinatorial strategies and involves a screening step performed using mammalian cells. This method is universal and is in principle applicable to any species for which antibody sequence information is sufficient for designing primers for VH and VL cDNA amplification. The key property of HybriFree technology is its rapid and robust workflow: it takes approximately 10 days to go from source material collection to obtaining expression vectors for antigen-specific antibodies, together with sequence information regarding their VH-VL combinations. The method does not require the establishment of sterile B-cell cultures, single cell manipulations or specialized materials. Additionally, we have supplemented the method with an optional negative selection step to eliminate the recovery of antibodies directed to undesired off-targets.

## Methods

### Immunization of animals

#### Chicken

Six- to eight-month-old Hy-Line chickens were immunized by four (weeks 0, 2, 4 and 18) intramuscular (im) injections of 0.5 mg protein antigen in complete (initial immunization) or incomplete (subsequent immunizations) Freund adjuvant. An antigen boost was given intravenously (iv) 2 weeks after the final injection as 0.1 mg protein in PBS.

#### Mice

Four- to six-week-old female BALB/c mice were initially immunized intraperitionally (ip) with ~50 μg of protein antigen in complete Freund adjuvant (week 0), followed with 5 ip administrations (weeks 3, 7, 16, and 2 times in week 17) with the same amount of antigen in PBS.

#### Rabbits

Approximately 5-month-old New Zealand rabbits were initially immunized by 2 subscapular injections (one in each side of the body). The protein antigen, or virus-like particles (VLPs), was administered 4–5 times in amounts of 0.1-0.4 mg in complete Freund (first immunization only) adjuvant or in incomplete Freund adjuvant. The response was boosted by an intravenous injection of 0.1 mg protein antigen.

All of the procedures performed using animals were in accordance with the European Union directive 86/609/EEC and were approved by the Estonian National Board of Animal Experiments (No. 115, 07.09.2012; No. 87, 28.08.2007).

### Collection and preparation of spleen cells

After the confirmation of antigen-specific antibody response in egg yolk preparations (chickens; IgY) or in blood serum (mice and rabbits; IgG), spleens were collected 2–4 days after final immunization (i.e., the final boost). The animals were anesthetized, and cardiac puncture was used to collect blood. The spleen was removed and stored on ice until treatment (within one hour). For the preparation of cell homogenate, the spleen was homogenized in ice-cold PBS using a 40 μm cell dissociation sieve. The material was collected in a 50 ml cell culture tube and precipitated by centrifugation at 300 × g for 5 min at 4 °C. The cells were re-suspended in 50 ml of ice-cold PBS and centrifuged again. Finally, the precipitated cells were suspended in 5 ml ice-cold freezing medium (heat inactivated fetal bovine serum + 10 % DMSO), distributed into cryovials (1 ml per tube), and slowly frozen to −80 °C. For longer storage, the tubes were transferred into liquid nitrogen after 4–5 days.

Preparation of the cells for capturing: The frozen spleen cell suspension was thawed and transferred into 10 ml of RPMI1640 cell culture medium at ambient temperature. The cells were collected by centrifugation (200–300 × g, 5 min, room temperature), re-suspended in 10 ml of RPMI1640 supplemented with penicillin/streptomycin and 10 % of heat inactivated fetal bovine serum (rabbit or mouse splenocytes) or chicken serum (chicken splenocytes). The cells were seeded into a 100 mm cell culture dish and incubated ~1 h at 37 °C in a 5-8 % CO_2_ atmosphere. Then, free-floating cells were carefully collected, and the viability of the cells was evaluated using trypan blue exclusion. The cells were sedimented by centrifugation and re-suspended in the capture medium (RPMI1640 supplemented with 0.5 % BSA and 0.1 % NaN_3_). The chicken splenocyte preparations contained a high amount of erythrocytes; therefore, an additional Optiprep™ (60 % iodixanol, Axis-Shield PoC AS, Norway) gradient purification step was included by the sedimentation of lymphocytes along a density barrier [15] before re-suspension in the capture medium.

Capturing: MaxiSorp™ surface 96-wells (Thermo Fisher Scientific, US) were coated with the antigen (20 μg/ml in PBS, 4 °C, overnight) and blocked for 1–2 h with 2 % BSA in PBS. One hundred microliters of cell suspension containing 2 x 10^4^ live cells in capture medium were loaded into a single well. The plate was centrifuged (200 × g, 5 min) and incubated for 45–60 min. The medium was discarded, and loosely attached cells were removed by washing 4–5 times with PBS, each time pipetting gently triturating (3–4 times) at the edge of the well. Finally, the plastic-bound cells were lysed in the wells using 200 μl of TriReagent® (Molecular Research Center, US) and 1–2 μg of yeast tRNA (Life Technologies, US) carrier added to each well. Total lysate of the human cell line U2OS was used as a carrier rather than tRNA in some experiments using chicken splenocytes.

### RNA isolation and cDNA first-strand synthesis

The total RNA isolation was performed as recommended by the TriReagent® manufacturer. Material from 2 (mouse, rabbit) or 8 (chicken) wells was pooled together during the isolation. The final RNA samples were dissolved in 8 μl of nuclease-free water and subjected to cDNA first-strand synthesis using the SuperScript® III First-Strand Synthesis System according to the manufacturer’s instructions (Life Technologies, US). This preparation yielded 20 μl of cDNA reaction per sample.

### PCR amplification of VH and VL regions

PCR reactions were performed in 50 μl for a total of 35–40 cycles using Phusion Green Hot Start II High-Fidelity DNA Polymerase (Life Technologies, US) with pre-optimized conditions for each reaction. The primers used for the amplification of antibody VH and VL regions were designed to maximally cover the variability of the VH and VL sequences. Primer pairs used for the amplification of chicken IgY regions were designed based on published data [16]. The mouse primer cocktails used for the amplification of VH and VL_κ_ sequences were designed based on published data [17] as well as V- and J-region cDNA sequences available in the international ImmunoGeneTics information system® (IMGT®) web resource [18]. For the construction of scFv fragments, rabbit VH and VL primer cocktails were designed and prepared using previously published data [19]. The sequences of the primers are listed in Additional file 1. The proprietary VH and VL primers were used for the construction of libraries expressing intact rabbit IgG molecules were designed using rabbit sequences stored at IMGT® [20]. For cloning purposes or forming the coding sequence of the flexible linker (GGGGS)_3_ between the VH and VL domains, an additional 20–23 nucleotides were added to the 5’ ends of primers.

### Circular Polymerase Extension Cloning and plasmid DNA isolation

The restriction- and ligation-independent Circular Polymerase Extension Cloning (CPEC) method [21] was used for library construction by the in-frame-directed cloning of amplified VH and VL regions into the pQMCF mammalian expression plasmid (Icosagen Cell Factory, Estonia). Briefly, the variable domain PCR products and linearized/dephosphorylated vector fragment(s) were separated and purified from a TAE-agarose gel. Approximately 100 ng of the vector together with inserts (~2 times molar excess of each) were used in the 20 μl CPEC reaction with Phusion High-Fidelity DNA Polymerase (Life Technologies, US). Totally 25 cycles of denaturation, annealing and extension were performed according to the the polymerase manufacturer’s instructions. Five microliters from the reaction were used for the transformation of competent TOP10 F’ or DH5α strain *E. coli* cells. Approximately 1/10 of the transformation mixture was used for the direct inoculation of 2 ml of selective carbenicillin-containing liquid growth medium, followed by the extraction of plasmid DNA (i.e., the library pool) from overnight culture. Another part of the transformation mixture was plated onto carbenicillin-containing solid medium to obtain individual clones. The bacterial clones were amplified in 0,75 ml of liquid medium, and plasmid DNA mini-preparations were purified using a Zyppy™-96 Plasmid Miniprep kit (Zymo Research, US) or a FavorPrep™ 96-Well Plasmid Kit (Favorgen Biotech Corp., Taiwan) according to the manufacturer’s instructions.

### Cells, transfection and sample collection for mammalian screening

The Chinese hamster ovary (CHO-S from Thermo Fisher Scientific, US)-derived cell line CHOEBNALT85 (Icosagen Cell Factory, Estonia) was grown in serum-free chemically defined medium and was used for antibody screening. This cell line expresses EBV EBNA1 protein and mouse polyomavirus large T protein and is specifically designed for the prolonged and high level production of proteins in association with pQMCF vectors (USPTO Patent No: 7,790,446). The cells were transfected using chemical transfection Reagent 007 (Icosagen Cell Factory, Estonia) according to the published protocols [22]. One microgram of plasmid DNA was transfected in 6-well plate format for analyzing library pools, and approximately 0.2 - 0.5 μg DNA per sample was used in a high-throughput 96-well plate transfection for screening individual clones. Seventy-two hours after transfection, the supernatants were collected for analysis. When necessary, scFv-Fc or human IgG1 concentrations in the samples were determined using FastELISA for Human IgG quantification (RD Biotech, France).

### ELISA

The ELISA plates (Nunc™ MaxiSorp™, Thermo Fisher Scientific, US) were coated at 4 °C overnight with antigen solution (2–5 μg/ml) or VLP suspension (20 μg/ml) in PBS, washed with washing solution (PBS containing 0.05 % Tween 20), and incubated 1–2 h with blocking solution (PBS containing 2 % BSA and 0.05 % Tween 20) at room temperature. After washing twice, the culture supernatants (diluted in blocking solution, if necessary) were incubated 1–2 h at room temperature. After washing 4 times, a second incubation was performed with goat anti-human IgG (for scFv-Fc) or anti-rabbit IgG antibody conjugated with HRP (LabAs, Estonia). The signals were developed with TMB VII substrate (Biopanda Diagnostics, UK). The reactions were stopped by the addition of 0.5 M H_2_SO_4_, and absorbance values were measured at 450 nm.

### Sequence analysis

Protein sequences of identified antibody VH and VL were analyzed by exhaustive pairwise global alignments and the progressive assembly of alignments using Neighbor-Joining phylogeny for similarity determination. This was done using Clone Manager Professional (Scientific & Educational Software) and BioEdit Sequence Alignment [23] software. Complementarity determining regions (CDRs) in VH and VL amino acid sequences were determined using ref. [16, 18, 20].

## Results and Discussion

### Description of the HybriFree technology

**Fig. 1:**
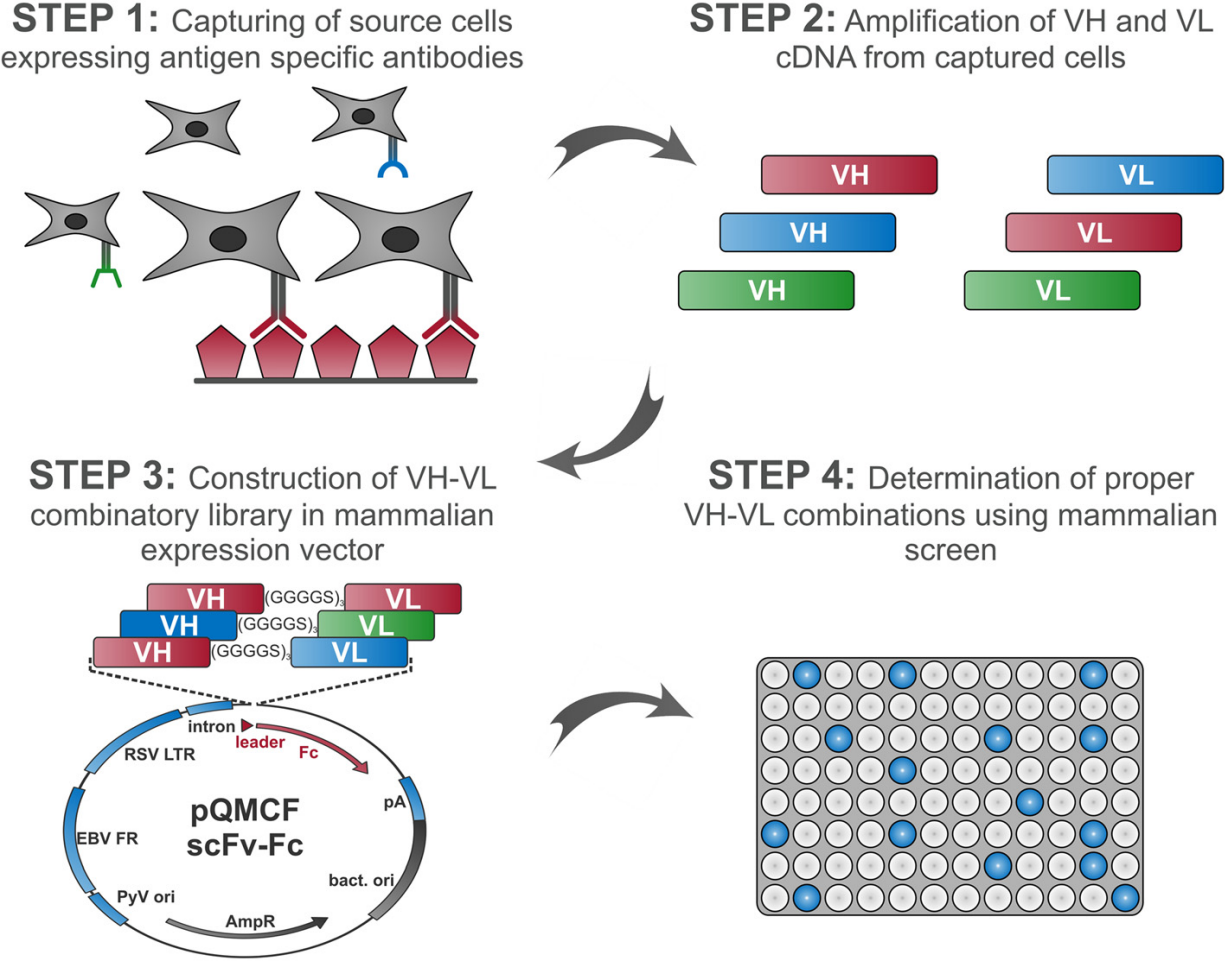
General workflow of the HybriFree method. The key steps include (STEP 1) the enrichment of source material by capturing cells producing specific antibodies; (STEP 2) the amplification of VH and VL cDNA; (STEP 3) construction of a combinatorial VH-VL library in scFv-Fc (or IgG) expression plasmid DNA format; and (STEP 4) screening proper VH-VL combinations after expression of MAbs in mammalian cells

The overall HybriFree procedure is illustrated in Fig. 1. Its key steps include: (i) the enrichment of source material via capturing antibody producers by exposing them on the antigen-loaded solid matrix; (ii) the amplification of VH and VL coding cDNAs; (iii) the construction of a combinatorial VH-VL library in mammalian expression plasmid DNA format; and (iv) screening the proper VH-VL combinations after antibody expression in mammalian cells. Because screening is usually performed by the transfection of individual DNAs into mammalian cells in 96-well format, hundreds rather than thousands of clones can be easily screened in a single round. To ensure that appropriate VH-VL combinations are discovered from a limited number of clones, it is crucial to maintain the diversity of the library in a narrow range. Ideally, the library should include only the VH and VL regions that are found in the antibodies recognizing the antigen of interest. Here, this diversity limitation is achieved by the functional selection of source cells before the VH and VL regions are isolated. This is ensured by capturing the source cells on the solid surface (e.g., ELISA plate well) coated with the antigen (step 1, upper left in Fig. 1). The technique is historically referred to as “lymphocyte panning” (reviewed in ref. [24]), and the capture is performed via interactions between the surface immunoglobulin (sIg) molecules of the B-cells [25] and the immobilized antigen. For example, lymphocyte panning has been used to establish retained B-cell cultures that have been used as a source of VH and VL sequences [26, 27]. Microwell array chip assay or fluorescence-based methods have also been used for the identification and isolation of cells secreting antibodies with desired properties (reviewed in ref. [28]). However, to our knowledge, this technique is predominantly used for the isolation of viable antigen-specific cells for hybridoma generation and/or in vitro culturing. In contrast, by the HybriFree method, the captured antigen-specific cells are immediately subjected to lysis, and therefore the viability of the yielded cells and selecting the conditions suitable for B-cell culturing do not pose issues. Instead, we have optimized cell capture conditions for HybriFree technology.

The captured source cells are subjected to RNA isolation, cDNA preparation, and the amplification of VH and VL cDNAs (step 2, upper right in Fig. 1), followed by the construction of a combinatorial library in a mammalian expression vector (step 3, lower left in Fig. 1). Using high-throughput techniques, the plasmid DNA was prepared from single bacterial colonies grown in 96-well plates and transiently transfected into mammalian cells. Thereafter, antigen-specific antibody production is evaluated for each clone (step 4, lower right in Fig. 1). Finally, VH and VL cDNAs are sequenced for the selected positive clones. The whole process takes no more than 10 days.

Additionally, we introduced an optional pre-adsorption step to avoid the undesired cross-reactivity of the developed MAbs. In brief, the source cells are treated with an excess of off-target protein prior to capturing with desired target antigen. In this way, all binding sites on the cells that are cross-reactive with undesired off-targets are saturated and cannot bind to the target antigen anymore. In the following sections, this method is described in detail with proof-of-principle examples provided regarding the isolation of MAbs from rabbit, mouse, and chicken. Applying the pre-adsorption step for increased selectivity is also illustrated.

### Capturing antigen-specific B cells

We have optimized the capturing of antigen-specific B cells in antigen-coated 96-wells (MaxiSorp™ surface) using a non-sterile crude suspension of intact spleen cells from different animals. However, the removal of plastic-adherent cells (e.g., macrophages) from lymphocytes during the first incubation step was found to increase the capture of antigen-specific lymphocytes as previously described [29].

We have most carefully optimized the number of source cells used per single capture reaction. The number of bound cells must be sufficient for the robust one-step amplification of the VH and VL regions. Even if the amplified VH and VL regions correspond to only those antibodies that bind to the target antigen, it is necessary to maintain the diversity of library within limits suitable for a mammalian screen. To this end, we have found that 2–6 x 10^4^ mammalian splenocytes and 8–16 x 10^4^ chicken splenocytes per cDNA sample is optimal. We have determined that to increase the variability of the recovered MAbs, setting up more capturing reactions/well is preferable to increasing the cell number per well. We also observed that ~45 min capture time in ambient temperature is sufficient for cells to bind to the antigen, without being so long that major degradation of cellular mRNA without *de novo* synthesis becomes an issue.

**Fig. 2:**
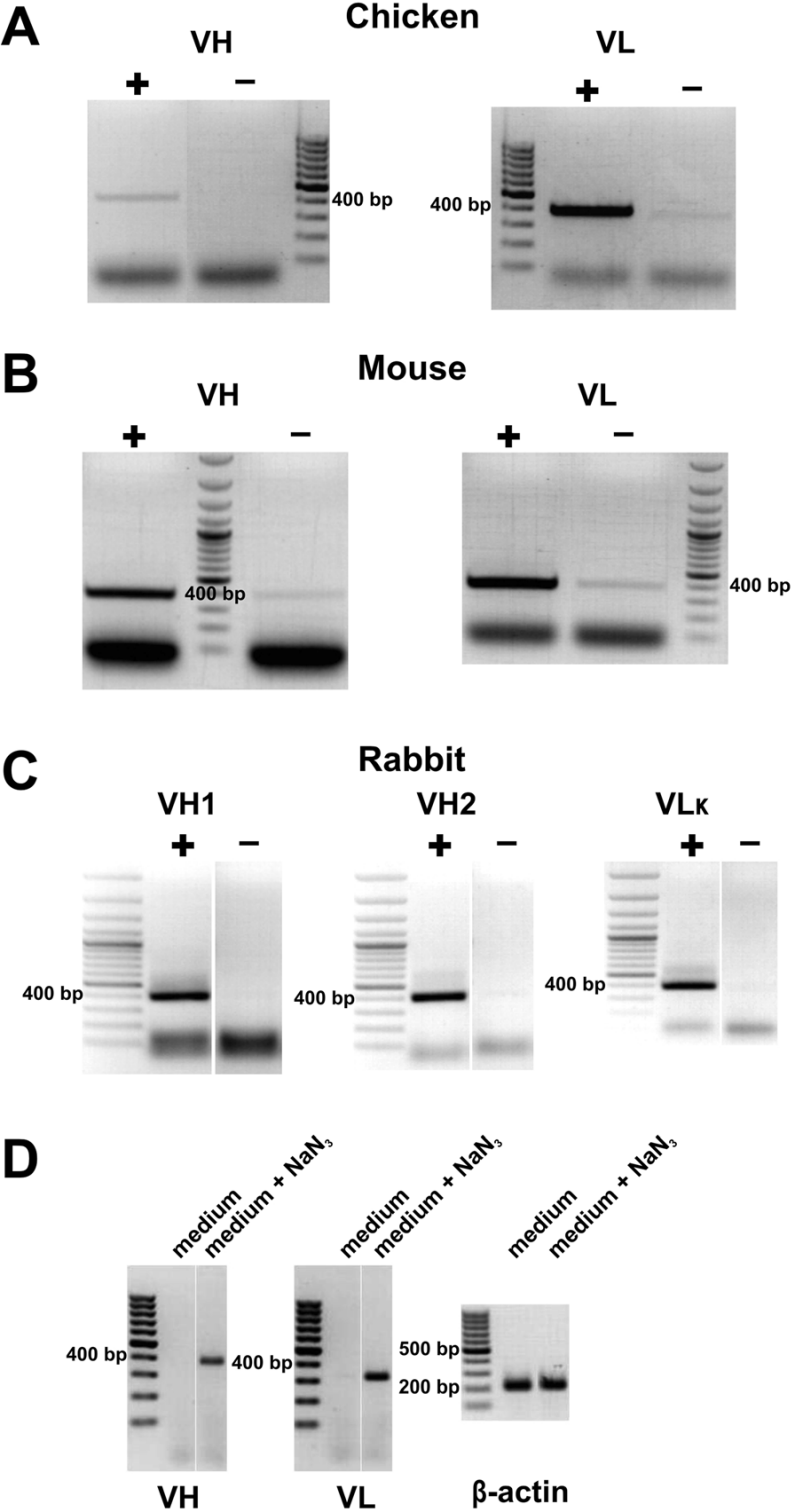
Amplification of VH and VL cDNA from spleen cells captured on an antigen-coated surface. cDNA was prepared from cells captured on the antigen-coated surface (+) and from control wells coated with BSA (−). **a** Splenocytes from chicken immunized with human DNase I protein. **b** Spleen homogenate from mouse isolated after immunization with human artemin protein. **c** Spleen homogenate from rabbit immunized with GST-E2C fusion protein. **d** Improvement of the VH and VL product yield by NaN_3_. Chicken splenocytes were captured in capture medium with or without 0.1 % NaN_3_, as indicated. Amplification from human β-actin cDNA was performed (209 bp product from spliced mRNA) as equal amounts of human cell lysate were used as a carrier during RNA isolation

The capturing process is illustrated by examples using chicken immunized with human DNase I protein, mice immunized with human artemin protein, and rabbit immunized with an HPV18 E2 protein domain fused with GST (GST-HPV18 E2C). The success of antigen-specific cell capture was first suggested by the agarose gel electrophoresis of the ~400 bp VH and VL amplification products, as shown in Fig. 2a-c. In lanes marked (+), the cells were captured by the relevant target antigen. As a negative control, identical capturing procedures were performed using wells coated with the non-relevant protein (lanes marked (−) in Fig. 2). Comparing the signal intensities of PCR products between the (+) and (−) lanes in Fig. 2, it is obvious that for all source animals, the VH and VL products were amplified much more efficiently from (+) cells. However, the capture selectivity is somewhat dependent on the particular target antigen. High background levels we have sometimes met are probably caused by the binding of the spleen cells to the target antigen via mechanisms other than antibody-antigen interaction.

Supplementing the capture medium with 0.1 % NaN_3_ improved the yields of amplified PCR products, especially for chicken splenocytes and/or when cell capture was initially performed at a higher temperature. This is illustrated in Fig. 2d by VH and VL amplification from splenocytes collected from chicken immunized with mouse CD48 protein. The cells were captured on an antigen-coated surface at 37 °C using RPMI1640 + 0.5 % BSA or the same medium supplemented with 0.1 % of NaN_3_ as the capture medium. The yield of the VH and VL products was much higher for reactions supplemented with NaN_3_. The success of RNA isolation and cDNA synthesis was confirmed by amplification from human β-actin-spliced mRNA (an equal amount of human cell line lysate was used in both samples as a carrier in RNA isolation). We speculate that the favorable impact of NaN_3_ may be caused by the blocking of antibody internalization and/or mRNA degradation during capture.

### ScFv-Fc library construction

**Fig. 3:**
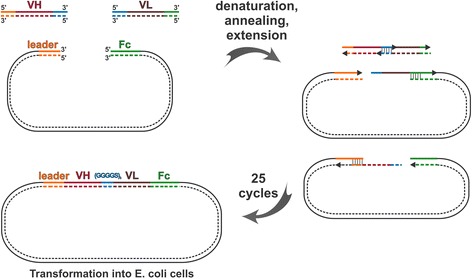
Generation of scFv-Fc library by CPEC. Amplified VH and VL fragments are cloned in frame into a vector between secretion leader and Fc region cDNAs using overlapping regions added at the ends of the PCR products. The overlapping sequences added to variable domains formes a flexible linker (GGGGS)_3_ between VH and VL. In each of 25 CPEC cycle, the denaturated inserts and vector are hybridized and extended until completing full circles. The plasmid library is propagated and recovered by transformation into competent E. coli cells

Next, VH-VL combinatorial libraries were constructed from amplified VH and VL regions (shown in Fig. 2) by the CPEC technique [21]. For rabbit splenocytes, two PCR reactions (VH1 and VH2) were used for better coverage of VH cDNA (Fig. 2c). Thus, two separate cloning reactions were performed by combining the VH1 product or VH2 product with the VL_κ_ product. As illustrated in Fig. 3, during the CPEC reaction between the 3 fragments (vector, VH, VL), pQMCF scFv-Fc vectors were formed via random combination of VH and VL fragments and in-frame joining of the scFv cDNA 5’ and 3’ ends with mouse immunoglobulin heavy chain secretion leader peptide cDNA and human IgG1 Fc region cDNA, respectively. The CPEC reaction is performed by annealing and extending overlapping regions at the ends of joined DNA fragments. Thus, during the PCR primer design we added 20–23 nt. sequences from the 3’ end of leader peptide cDNA and the 5’ end of the Fc region to the 5’ end of VH and the 3’ end of VL fragment, respectively. Similarly, using overlapping sequences added during the PCR, coding sequence of a flexible linker (GGGGS)_3_ is created between the VH and VL cDNAs. This enables CPEC to join 3 fragments into the scFv expression vector (step 3 in Fig. 1) without the addition of nucleotides between the vector and the insertions.

Library pool samples (a mixture of all library members) and single clone DNA minipreparations (using a convenient high-throughput 96-well method) were prepared from *E. coli* cells directly transformed with CPEC reaction product. PCR analysis of bacterial colonies showed the high efficiency of the cloning, and routinely >90 % of CPEC clones contained directed VH-VL insertion in the vector (data not shown).

### Mammalian cell screening of antigen-specific scFv-Fc molecules

In the examples provided below, we used the CHO-derived cell line CHOEBNALT85 for screening. These cells grow in serum-free chemically defined medium and ensure high transfection efficiencies in a variety of scales, including a 96-well high-throughput format. In association with the pQMCF vector, CHOEBNALT85 cells ensure relatively high transient production levels of IgG-like molecules (typically tens of micrograms per milliliter at 72 h time-point using a 2 ml culture scale). Moreover, we routinely use the same cells for recombinant antibody production at larger scales. Using the same cells for screening and production decreases the odds of subsequent problems with productivity. However, any mammalian cell line that can be efficiently transfected with plasmid DNA expression vector is usable for screening by this method. In the examples described here, the expression of scFv-Fc was controlled using the Rous sarcoma virus long terminal repeat promoter.

**Fig. 4:**
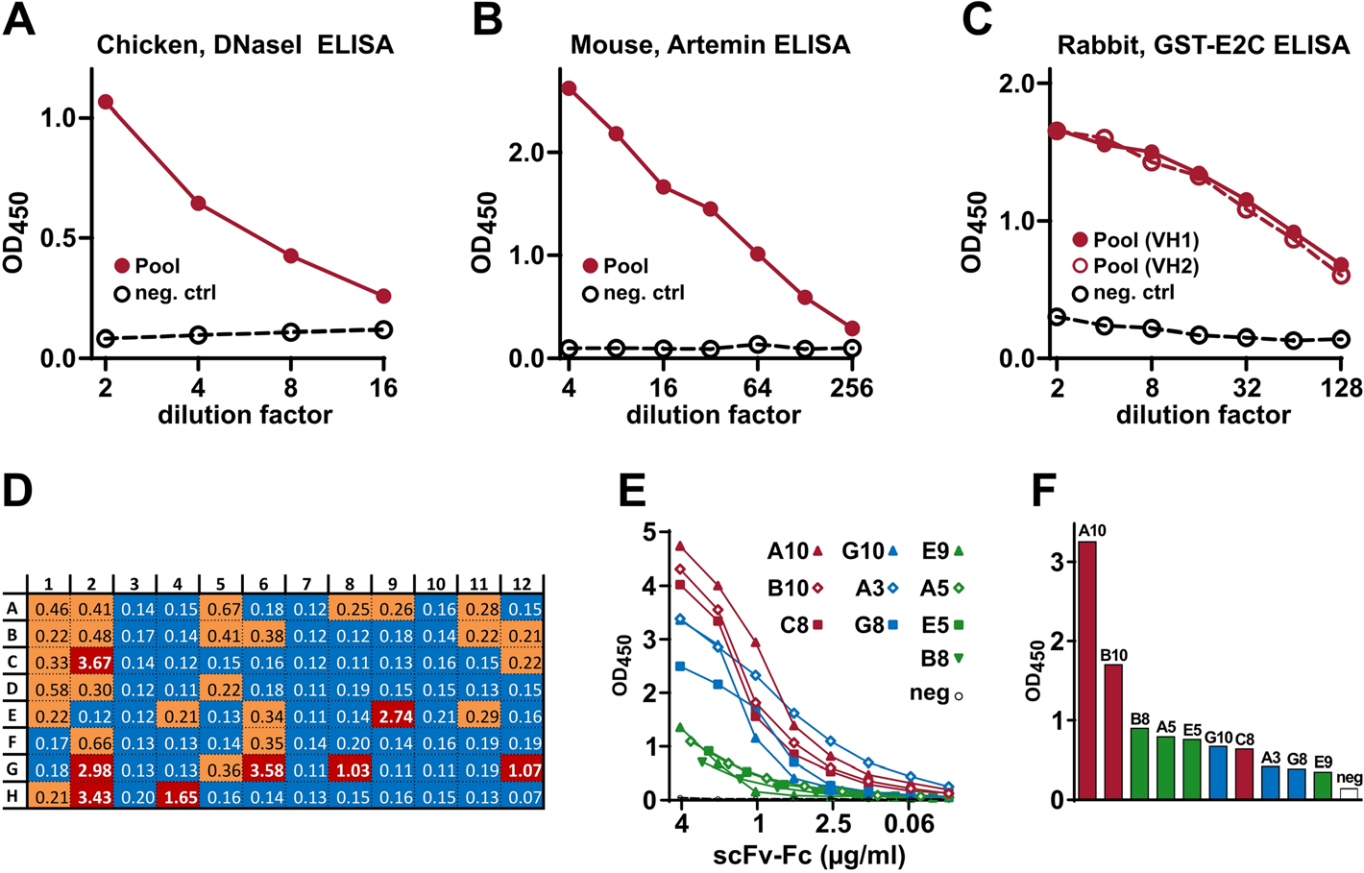
Exemplary ELISA analyses of constructed scFv-Fc antibody libraries. **a**-**d** The graphs represent the ELISA results of serial dilutions of 72 h time-point supernatants from CHOEBNALT85 cells transfected with 1 μg of HybriFree library pool DNA from the following captured cells: (**a**) chicken immunized with human DNase I; (**b**) mouse immunized with human artemin; and (**c**) rabbit immunized with GST-E2C protein. The irrelevant scFv-Fc vector was used as the negative control in each transfection. **d** ELISA results of single clone screening of the rabbit GST-E2C library pool shown in panel **c**. Wells G12 and H12 were transfected with library pool and negative control DNA, respectively. The clones gave highly positive OD signals ≥ 15 times over the negative control are highlighted in red. The clones with the signals 3–10 times higher than the negative control have orange highlighting. **e** Normalized artemin ELISA of 10 anti-artemin clones obtained from the immunized mouse (library pool ELISA results are shown in Fig. 3b). **f** Absorbance readouts of the same clones used in initial screening

The efficiency of the antigen-specific MAb reconstruction from VH and VL combinations was initially analyzed via the transfection of library pools. The DNA was transfected into CHOEBNALT85 cells, and 72 h later the culture supernatants were assessed by ELISA for the secretion of antigen-recognizing scFv-Fc molecules. This is illustrated with the pools created by extraction of plasmid DNA (from overnight culture of competent E.coli cells transformed with the CPEC reaction with VH and VL products from chicken, mouse or rabbit showed in Fig. 2. As seen in Fig. 4a-c, positive signals qualitatively different from the controls were observed in all cases, suggesting the expression of antigen-specific MAbs in the libraries. As a next step, individual MAbs with specific binding were identified by screening plasmid DNA minipreparations derived from single clones. Using the high-throughput methods, the mini-preparation plasmid DNA was isolated from individual bacterial clones from the CPEC and transfected into CHOEBNALT85 cells. One or two 96-well plates (including appropriate positive and negative controls) were analyzed per library, usually 48 or 72 h post-transfection We arbitrarily defined positive signals as ELISA OD readouts at least 2–3 times over the negative control (empty expression vector or expressing a non-relevant scFv-Fc). However, especially in 72 h time-point, the positive signals typically qualitatively discriminated from the negative samples by a readouts at least 10 times higher (Fig. 4d). The efficiency of screening (percentage of positive hits) in our experiments has varied between 7 % and 60 % for different antigen and animal combinations. The net results of efficiency and outcomes of HybriFree experiments involving different animals, library types and antigens are summarized in Table 1.

**Table 1:** Hit rates (proportion of antigen recognizing clones) achieved from positive library pools in different HybriFree experiments

Source	Library	Antigen	Pos./all (%)	Comments
Chicken	scFv	Persephine	18/118 (15 %)	After B-cell capturing as described here, the screening was done using E. coli based system
		GFRα1	71/140 (50 %)	
	scFv-Fc	hDNase	3/43 (7 %)	
		hNGF	3/23 (13 %)	
		HPV18 E2	7/93 (8 %)	Full-length protein was used for immunization but the B-cell capturing and screening were performed using only C-terminal domain (one-third)
Mouse	scFv	Persephine	19/190 (10 %)	After B-cell capturing as described here, the screening was done using E. coli based system
	scFv-Fc	Artemin	12/78 (15 %)	
		hNGF	7/24 (29 %)	
Rabbit	scFv-Fc	hCD48	7/46 (15 %)	
		HPV18 E2N-GST	13/93 (14 %)	
		HPV18 E2C-GST	16/188 (9 %)	Summarized results obtained from 2 rabbits
		mCD48	80/186 (43 %)	
		Ebola VLP	25/94 (27 %)	
		Ebola GP	104/234 (44 %)	Summarized results obtained from 3 experiments
	IgG	Ebola GP	11/46 (24 %)	
		mCD48	55/94 (59 %)	see section “Screening natural intact IgG molecules” for more details
		hBDNF	43/125 (34 %)	

High-throughput methods used for the preparation and transfection of plasmid DNA usually result in some variability in DNA concentration, and thus also in transfection efficiency. This can lead to differences in scFv-Fc expression/secretion level, also influenced by the actual DNA sequence (codon usage) of VH and VL cDNA. Thus, the differences in signals observed in 96-well screening do not necessarily reflect differences in the quality or affinity of the individual MAbs. Therefore, normalization is necessary for a head-to-head comparison of the selected MAbs. This is illustrated in Fig. 4 using 10 positive MAbs against human artemin screened from the mouse library. Normalized ELISA curves are shown in Fig. 4e, and the readouts of the same clones in an initial high-throughput screen are illustrated in Fig. 4f.

### Screening intact IgG molecules

**Fig. 5:**
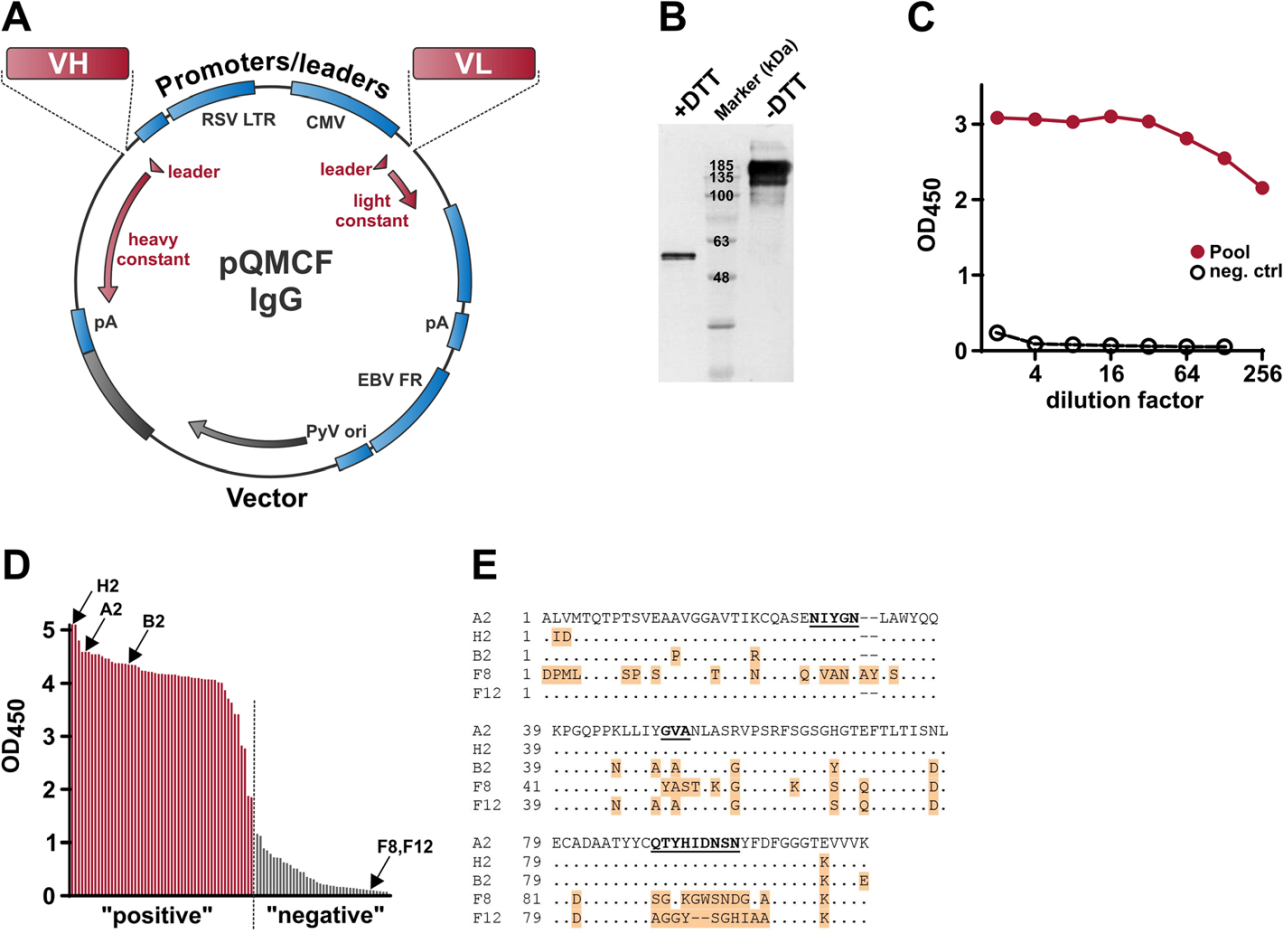
Construction and screening of intact IgG molecules. **a** The pQMCF IgG vector was constructed using single-step CPEC joining of 4 fragments: VH, VL, promoters/leaders and vector. The antibody heavy and light chains are expressed from the resulting vector as separate proteins that assemble naturally into IgG molecules secreted from mammalian cells. **b**. Western blot analysis of rabbit IgG secretion from CHOEBNALT85 cells transfected with pQMCF IgG library pool DNA constructed from VH and VL regions from a rabbit immunized with mouse CD48 protein. Goat polyclonal antibody against rabbit IgG heavy chain was used for the detection of free heavy chain in reduced sample conditions (DTT+) and of the assembled IgG molecule in non-reduced (DTT-) sample. **c** Mouse CD48 ELISA results obtained using serial dilutions of the same sample of library pool transfection media as primary antibody. **d** Distribution of positive and negative clones obtained from the screening of the library pool showed in the panel **c**. Three positive and two negative clones selected for sequencing are indicated. **e** Alignment of VL domain amino acid sequences of the positive and negative clones. CDR regions are underlined

Isolation of antigen-specific VH and VL pairs via scFv-Fc screening provides enough information for the construction of expression vectors necessary for the recombinant production of the IgG antibodies from heavy and light chains expressed in the cell as separate polypeptides. However, in this case, cDNA re-synthesis (by PCR or gene synthesis), changing the expression vector, and additional cloning steps are necessary. This makes the process cumbersome and time-consuming. Thus, we modified our method by screening the VH and VL combinations so that intact IgG molecules are initially formed rather than scFv-Fc antibodies. To this end, the CPEC strategy was modified to enable the directed in-frame joining of the 4 fragments (VH, VL, promoters/leaders, and vector). Here, the VH and VL are both exactly joined with the secretion leader peptide cDNA at the 5’ end and with constant domain cDNA at the 3’ end. The reaction creates just natural joining between variable and constant domain in the IgG heavy and light chain, respectively. The final product resulting from the CPEC reaction is the pQMCF IgG vector, with separate expression cassettes for the IgG heavy and light chain, respectively (Fig. 5a). To ensure efficient directed CPEC assembly of 4 DNA fragments, the overlapping regions at the ends of the fragments were carefully optimized using synonymous replacements in cDNA encoding the leader peptide and constant region.

The modification in this method is illustrated by the development of rabbit MAbs against mouse CD48 protein. VH and VL fragments were created from spleen cells captured on mouse CD48-coated wells. The cloning reaction was performed using pQMCF IgG vector containing rabbit IgG heavy and light (kappa) chain constant region codon-optimized cDNAs. The newly formed IgG heavy and light chains were expressed under the control of the RSV LTR and CMV promoters, respectively. Initial restriction analysis of library pool DNA and colony PCR from 38 individual bacterial colonies indicated 100 % efficiency of pQMCF IgG vector assembly from the 4 fragments (data not shown). The Western blot analysis of the culture medium sample from library pool DNA-transfected CHO85EBNALT cells confirmed the predominance of intact rabbit IgG molecules assembled from the separately expressed heavy and light chains (Fig. 5b). The analysis of the same library pool transfection medium sample in mouse CD48 ELISA demonstrated the presence of antigen-specific antibodies (Fig. 5c). Finally, the screening of 95 clones in CHO85EBNALT cells resulted 39 % of clones with ELISA readouts 15–45 times over negative control and considered as “positive” (Fig. 5d).

Some positive (H2, A2, B2) and negative (F8, F12) clones (readouts indicated in Fig. 5d) were selected for sequencing of heavy and light chain cDNAs. All sequenced clones had essentially identical VH sequence, suggesting that the binding with the antigen was mainly determined by the VL domain. In this, the differences between the sequences of positive and negative clones were observed, most recognizable in CDR3 region (Fig. 5e). We have been observed that very high percentage of positive clones in the screening often associate with the limited number of different VH or/and VL sequences that are combined in the library. It may indicate that only few best binders have remained associated with the antigen coated surface during the repeated washing steps in B-cell capturing. Alternatively, particular cDNA product among others (e.g. this one provides most optimal template for the primer mix during the initial rounds of PCR) may be preferrably amplified. Irrespective of the excact mechanism, we have determined that increasing the variability of the recovered MAbs can be achieved by setting up more capturing reactions. This is clearly preferable to increasing the cell number per well.

### Selective capturing using the pre-adsorption of source cells

**Fig. 6:**
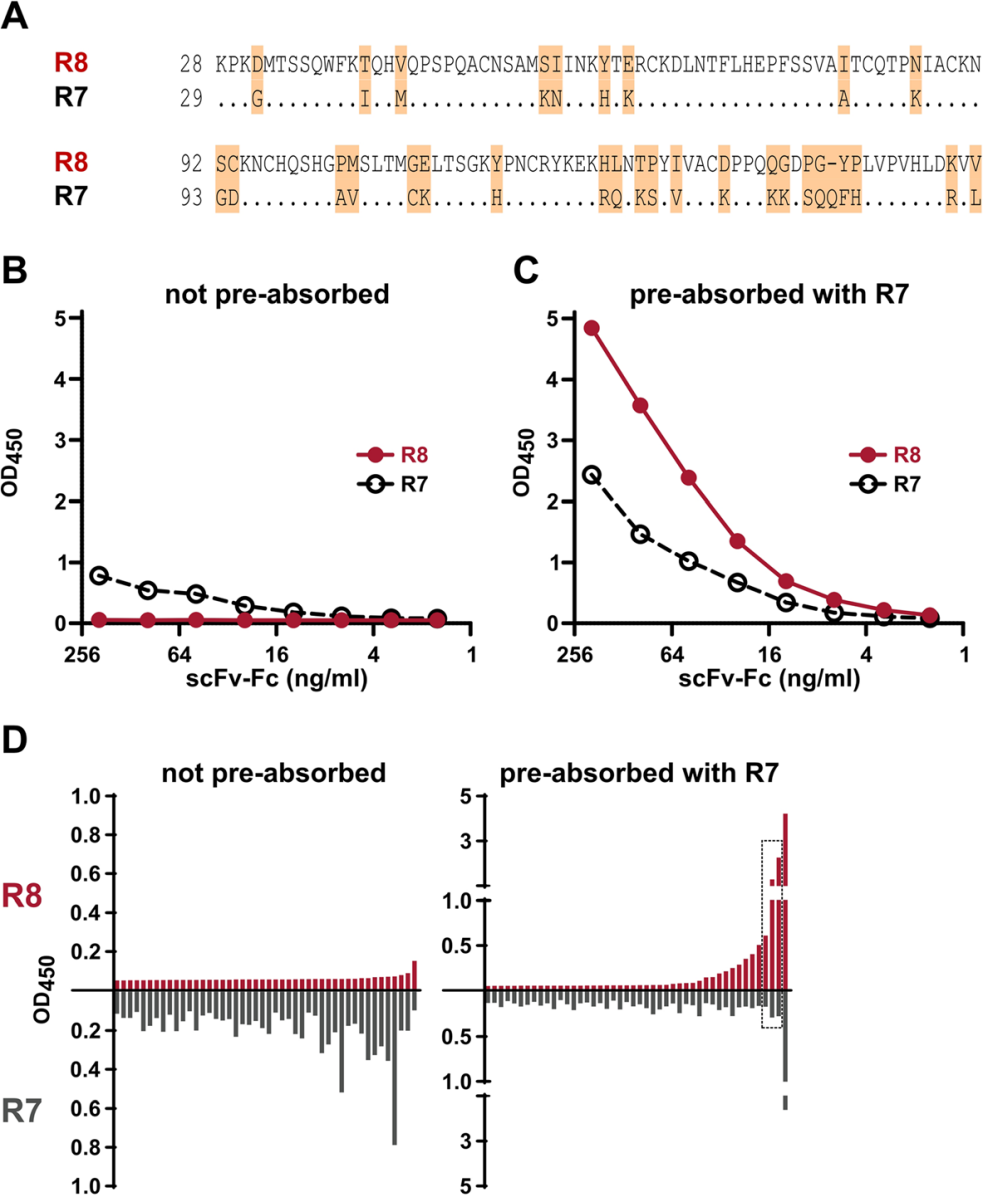
Selective development of non-cross-reactive antibodies using a pre-adsorption step. **a** Global alignment of the mature protein sequences of human ribonuclease 8 (R8, UniProt ID: Q8TDE3) and ribonuclease 7 (R7, UniProt ID: Q9H1E1). **b** ELISA assay result obtained using supernatants of CHOEBNALT85 cells transfected with 1 μg of library pool DNA derived from splenocytes captured without a pre-adsorption step. **c** The same ELISA assay using the library pool from splenocytes captured using the pre-adsorption step with R7 protein (100 μg/ml). **d** The distribution of R7-reactive (R7+), R8-reactive and only R8-reactive (R8+/R7-) scFv-Fc clones retrieved from the pre-adsorbed and control libraries, respectively

We are interested in the development of reagents for immunoassays usable for the measurement of protein antigens in different test samples. Thus, there is a need for MAbs that recognize specific target proteins or even protein isoforms but not off-target antigens sharing cross-reactive or even identical epitopes. We have observed that MAbs retrieved from immunizations with full-length proteins have unacceptable levels of cross-reactivity, and it is time-consuming to sort out the small number of “right” MAbs from the total population of recovered antibodies. Therefore, we have introduced an optional step during which the source cells are pre-adsorbed with an excessive amount of off-target antigen(s) prior to capturing with the desired target. In this way, all binding sites that are cross-reactive with the non-desired targets can be pre-saturated and cannot bind to the target antigen anymore. This is illustrated here by the development of MAbs against human ribonuclease 8 (R8) protein from spleen cells of an immunized rabbit. The goal was to obtain anti-R8 MAbs that have minimal cross-reactivity with the highly homologous human ribonuclease 7 (R7) (Fig. 6a); thus, a pre-adsorption step with R7 was included before cell capture. The R8-immunized rabbit spleen cells were captured in two ways: by using an R8-coated surface in capture medium as described in the section “Collection and preparation of spleen cells” above or by supplementing the medium with R7 protein (100 μg/ml) and including a short (15 min) pre-adsorption step in suspension before the cells were centrifuged to R8-coated wells. As illustrated in Fig. 6b, the library pool from cells not pre-adsorbed gave relatively low readouts. Most surprisingly, the responses were higher for R7 protein, and the readouts of R8 protein binding were just above background. This may indicate that dominant antigenic epitopes in the R8 protein exposed in vivo were not equally available to plastic bound antigen in vitro. In contrast, a potent anti-R8 response was detected in the library pool obtained using pre-adsorption with R7 protein. There was some response to the R7, but the titer was significantly higher for R8 (Fig. 6c). In two rounds, totally 92 individual clones were analyzed from both libraries for their binding to R8 and R7 protein, respectively. The results shown in Fig. 6d are in accordance with data from library pool analyses. The R7-reactive MAbs with weak readouts were detected in the library prepared without pre-adsorption (left diagram in Fig. 6d) but there were more R8-reactive than R7-reacive clones when the pre-adsorption was used (right diagram in Fig. 6d). Importantly, only from pre-adsorbed pool we found the desired MAbs that reacted with R8 and exhibited no or low cross-reactivity with the R7 protein (surrounded with box in Fig. 6d).

We propose that this pre-adsorption step is useful for screening antibodies from organisms immunized with complex antigens. For example, using the viral surface proteins pseudotyped VLPs rather than purified surface proteins alone for immunization generally gives better odds of recovering a virus-neutralizing antibody [30]. Using the same VLPs for capturing antigen-expressing cells as well as for screening yields opportunities to isolate and clone such antibodies. However, using VLPs for immunization usually results in a mixed response, including many MAbs specific to off-targets (e.g., directed to structural components of the VLPs). Thus, it may be useful to inhibit the recovery of the off-target antibodies by pre-adsorption of the antibody-expressing source cells with non-pseudotyped VLPs.

### Production, sequence analysis and applications of the developed MAbs

**Fig. 7:**
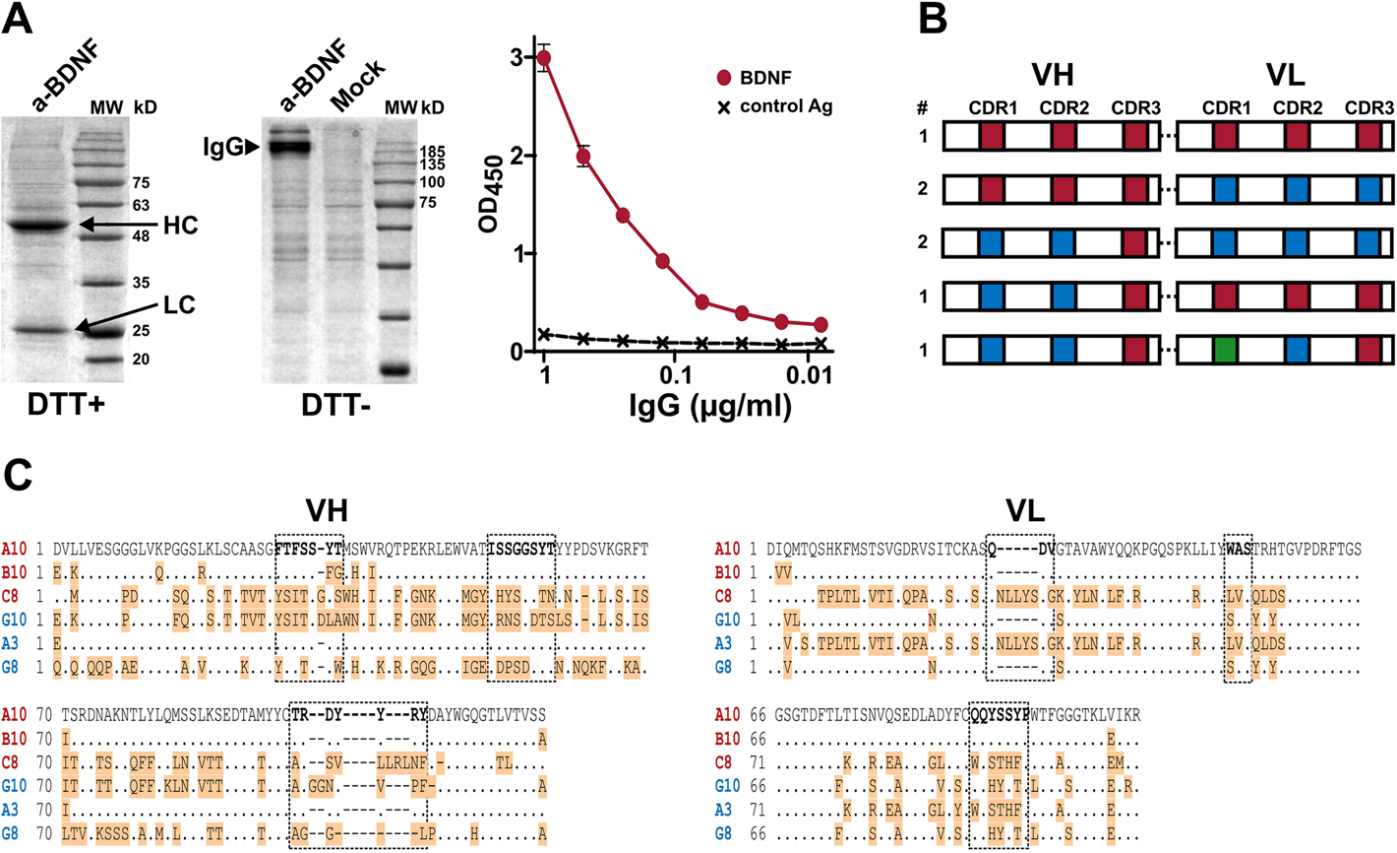
Production and sequence analysis of the obtained MAbs. **a** Coomassie staining of the supernatant (10 μl) collected after 10 days culture of CHOEBNALT85 cells transfected with the anti-BDNF IgG plasmid vector. Heavy and light chains and assembled IgG molecule are indicated in reduced (DTT+) and non-reduced (DTT-) sample conditions, respectively. Concentration dependent ELISA of the same antibody batch is represented by the diagram using coating with the BDNF or a non-relevant control protein. **b** Seven Mabs recognizing the C-terminal DNA binding domain of the HPV18 E2 protein were derived from immunized chicken. VH and VL protein sequence analysis revealed that their variable domains were formed using a limited number of CDR sequences. The same color in a particular CDR position (CDR1, 2 or 3 in the VH or VL, respectively) indicates an identical CDR sequence. The number of identified clones with this particular CDR pattern is shown at left (#). **c** Variable domain sequences of anti-artemin MAbs developed by the method from the spleen of immunized mice. The same color code is used as for the ELISA data of the same MAbs shown in the Fig. 4e and f. CDRs are surrounded by boxes

We developed the Hybrifree method to address shortcomings in our recombinant antibody isolation and production projects. Antibodies developed via screening in E. coli cells often showed low productivity or loss of activity after production in mammalian cells. To date, we have successfully used the HybriFree method for the isolation of antibody variable domain cDNA from mice, rabbits and chickens immunized with different viral and cellular antigens. The sequence information and/or expression vectors obtained by this method have been used for the establishment of larger scale transient production and the purification of scFv-Fc antibodies as well as intact IgG and IgY antibodies in CHO85EBNALT cells. Sometimes the DNA minipreparations used for screening can also be applied for immediate transient production of the selected antibodies in amounts sufficient for further characterization. For example, we found interesting MAb against human BDNF using the screening of IgG molecules (vector shown in Fig. 5a) with rabbit VH and VL domains fused with human IgG constant regions. Then the plasmid mini-preparation was used for transfection of serum-free culture of CHO85EBNALT cells. After 10 days approximately 40 ml of supernatant was collected that contained ~100 μg/ml of fully assembled anti-BDNF IgG with specific anti-BDNF activity confirmed by concentration curve in BDNF ELISA (Fig. 7a). The sequence analysis of developed MAbs has often revealed limited numbers of unique CDRs (CDR1-3 in VH and CDR1-3 in VL) that are differentially combined in the clones derived from a particular animal. For example, using 93 clones derived from chicken immunized with HPV18 full-length E2 protein, 7 MAbs identified by our method using only 1/3 of the of the protein (C-terminal DNA binding domain) for the B-cell capturing and screening. The sequence analysis of the MAbs revealed that the variable domains of these 7 antibodies were formed using a limited number of distinct CDR sequences: 1 sequence variant for VH-CDR3; 2 variants for VH-CDR1, VH-CDR2, VL-CDR2 and VL-CDR3; and 3 variants for VL-CDR1 (Fig. 7b). In general, higher variability was observed for mouse and rabbit antibodies. This is illustrated in Fig. 7c with variable domain sequences of mouse anti-artemin MAbs developed by the method (ELISA results are shown in Fig. 4e and f).

**Fig. 8:**
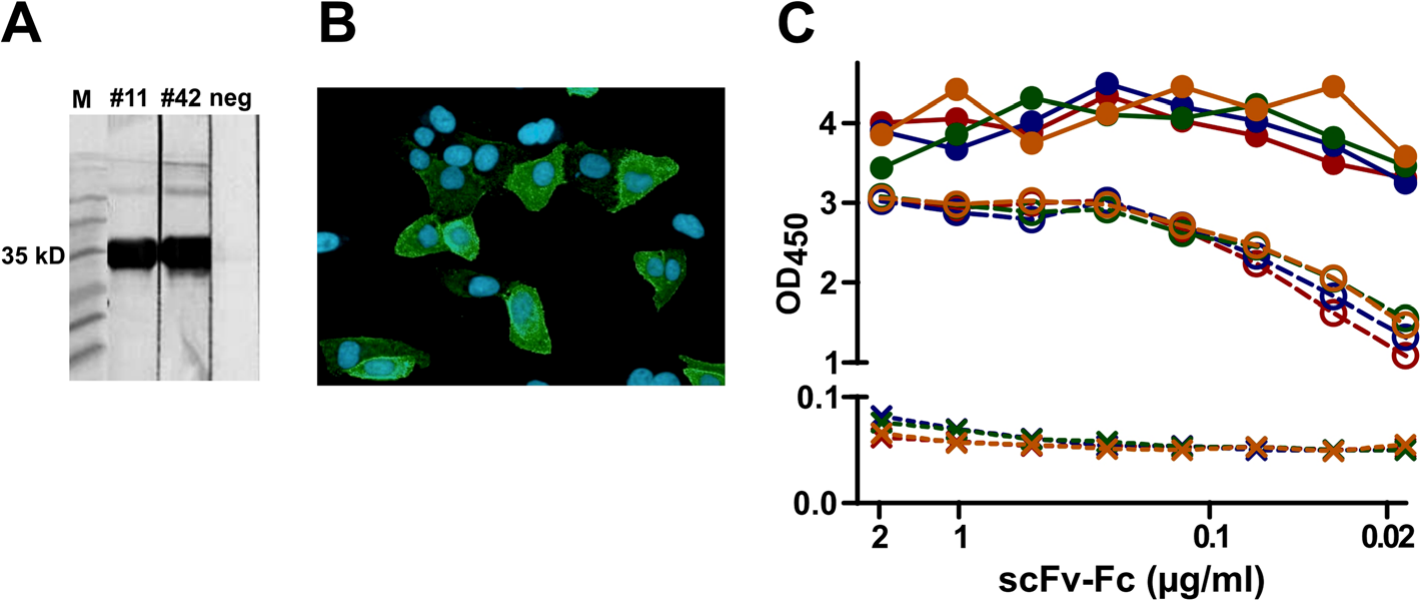
**a** Applications of the HybriFree MAbs. **a** Anti-human Dnase I IgY antibodies were re-constructed from developed MAb sequences from chicken. These were produced in CHO cells and can be used for the detection of a ~35 kD hDNase I band by western blot assay (the expression vector producing non-relevant IgY is used as a negative control). **b** GP-positive Ebola VLPs MAb derived from a rabbit immunized with Ebola virus VLPs serves as a sensitive and selective reagent for the visualization of Ebola virus glycoprotein (GP) expression (green) in human cells by immunofluorescence. Nuclei are counterstained with DAPI (blue). **c** ELISA assay with 4 anti-GP MAbs (each represented by different color) using purified GP (filled circles), GP-positive Ebola VLPs (open circles) or GP-negative Ebola VLPs (Xs) as a target

The variety of MAbs developed and characterized by their recognition of linear epitopes are useful for detection in assays such as immunoblotting (Fig. 8a) and immunofluorescence (Fig. 8b). These antibodies can also bind conformational epitopes and have potential applications in the development of products with biological activity. The usefulness of very specific MAbs developed by the method is also illustrated here with rabbit derived scFv-Fc molecules against Ebola virus GP. The reagent specificity is certainlt the issue for detection of the viral particles surrounded by the envelope derived from the host cell membrane that is studded with GP spikes. In Fig. 8c is shown of ELISA assay conducted with 4 anti-GP MAbs. Here the purified GP or GP-positive Ebola VLPs (both prepared in human 293 cells) was used as the target. Compared to purified protein (filled circles in Fig. 8c), the detection of membrane bound GP on the VLP (open circles in Fig. 8c) resulted in relatively lower but clearly positive readouts. However, non-specific binding to empty Ebola VLPs prepared without the GP component (marked by Xs in Fig. 8c) was not detected even at high concentrations of scFv-Fcs.

## Conclusions

We describe the novel HybriFree method for the development of monoclonal antibodies from an animal species for which coding sequence information of the antibody variable domain is available. This robust and rapid method includes the enrichment of source material by capturing specific antibody-producing cells for the construction of a combinatorial VH-VL library screened after expression in mammalian cells. The resultant expression vectors can be directly used to establish medium-scale transient production in mammalian cell factories without the need for additional cloning steps.

## Additional file

Additional file 1:
**Sequences of the primers used in VH and VL cDNA amplification for scFv-Fc construction.** This file contains primer sequences used in the study for amplification of chicken, mouse or rabbit VH and VL domain cDNAs. Additional 5’ nucleotides forming the overlapping region with the vector for CPEC cloning or the coding sequence of the flexible linker between the VH and VL domains are indicated. In addition, proportion of each primer in the cocktail is indicated. (XLSX 12 kb)
